# Supplementary material for: Content-rich biological network constructed by mining PubMed abstracts
Source: BMC Bioinformatics. 2004 Oct 8;5:147. doi: 10.1186/1471-2105-5-147 (PMC528731; doi:10.1186/1471-2105-5-147)
Supplement: Additional File 2 — The original results of the above study (non-essential files are deleted to keep the file size under the limit set by BMC bioinformatics). [file 1471-2105-5-147-S2.bz2 › chilibotAdditionalFile2/dip20/334ID8171323E3970/html/LTA_LTBR.html]

 


 **LTA** and **LTBR** 
  
Found 54 abstracts in PubMed, retrieved 20.  
 

 What does Google say? 
 PDF only 
| .edu only 

---

**Interactive relationship** (e.g. stimulation, inhibition, etc)

**Neutral relationship**- Mannheimia Pasteurella haemolytica A1 produces an extracellular leukotoxin  [ **LTA** ]  LKT that is reported to bind the beta 2 integrin CD11a CD18  [ **LTBR** ]  LEA 1 on ruminant leukocytes.  Ref: 11825601 Vet Immunol Immunopathol, 2002
- LT consists of soluble LT alpha  [ **LTA** ]  3, which binds to TNF R1 and TNF R2, and membrane LT alpha  [ **LTA** ]  1 beta 2, which binds to LT beta R  [ **LTBR** ] .  Ref: 12882833 Int Immunol, 2003
- These results indicate that the leukotoxin  [ **LTA** ]  binds to the beta 2 integrins on bovine leukocytes, very likely via CD18  [ **LTBR** ] .  Ref: 10481101 FEMS Microbiol Lett, 1999
- The lymphotoxin beta receptor  [ **LTBR** ]  LT beta R  [ **LTBR** ] , a member of the tumor necrosis factor TNF receptor family, plays a crucial role in lymphoid organogenesis by signaling through its functional ligand LT alpha  [ **LTA** ]  1 beta 2.  Ref: 11399522 Eur Cytokine Netw< MedlineTA>Eur Cytokine Netw, 1999
- Pre incubation of polymorphonuclear neutrophils with a monoclonal antibody specific for CD18  [ **LTBR** ]  reduced the cytotoxicity of the leukotoxin  [ **LTA** ]  to the cells.  Ref: 10481101 FEMS Microbiol Lett, 1999

**Non-interactive relationship** (e.g. studied together, co-existance, homology, etc.)

- This activity of LT depends on membrane LT alpha  [ **LTA** ]  1 beta 2 and not soluble LT alpha  [ **LTA** ]  3, since DA virus persisted and induced inflammatory demyelination in LT beta R  [ **LTBR** ] , but not TNF R1 or TNF R2, mice.  Ref: 12882833 Int Immunol, 2003
- LT alpha  [ **LTA** ]  deficient mice and LT beta R  [ **LTBR** ]  Fc transgenic mice were profoundly susceptible to murine CMV infection.  Ref: 11672543 Immunity, 2001
- We used mice lacking Peyer s patches and or lymph nodes because of lymphotoxin alpha  [ **LTA** ]  gene deficiency or treatment in utero with lymphotoxin beta receptor  [ **LTBR** ]  IgG and tumor necrosis factor receptor I 55 IgG fusion proteins.  Ref: 12466141 Am J Pathol, 2002
- The LT alpha  [ **LTA** ]  and LT beta R  [ **LTBR** ]  mice failed to mount a virus specific cytotoxic T cell response.  Ref: 12882833 Int Immunol, 2003
- Most lymphotoxin alpha  [ **LTA** ]  ltalpha and all lymphotoxin beta receptor  [ **LTBR** ]  ltbetar deficient mice are completely devoid of lymph nodes LNs.  Ref: 10586051 J Immunol, 1999
- Neither lymphotoxin alpha  [ **LTA** ]  nor lymphotoxin beta receptor  [ **LTBR** ]  expression is required for biogenesis of lymphoid aggregates or differentiation of natural killer cells in the pregnant mouse uterus.  Ref: 12603600 Immunology, 2003
- There was relatively little demyelination associated with inflammation in LT alpha  [ **LTA** ]  and LT beta R  [ **LTBR** ]  mice compared to susceptible SJL mice, suggesting the possibility that LT plays a role in mediating demyelination.  Ref: 12882833 Int Immunol, 2003
- Bovine CD18  [ **LTBR** ]  is necessary and sufficient to mediate Mannheimia Pasteurella haemolytica leukotoxin  [ **LTA** ]  induced cytolysis.  Ref: 12183553 Infect Immun, 2002
- Pre incubation of PMNL with monoclonal antibodies to CD11a and CD18  [ **LTBR** ]  that recognize alpha and beta chains of the LFA 1 integrin, a leukotoxin  [ **LTA** ]  receptor on PMNL, inhibited the cytolysis, but not the release of granule components.  Ref: 10863962 J Periodontal Res, 2000
